# Chemical Stability of the Lipid Phase in Concentrated Beverage Emulsions Colored with Natural β-Carotene

**DOI:** 10.1007/s11746-012-2194-8

**Published:** 2012-12-25

**Authors:** Arkadiusz Szterk, Marek Roszko, Ewa Górnicka

**Affiliations:** 1 Department of Functional Food and Commodities, Faculty of Human Nutrition and Consumer Sciences, Warsaw University of Life Sciences, 159 c Nowoursynowska, 02-776 Warsaw, Poland; 2 Department of Food Analysis, Institute of Agricultural and Food Biotechnology, 36 Rakowiecka str., 02-532 Warsaw, Poland

**Keywords:** Chemiluminescence, O/W emulsion, Emulsion stability, Natural dye

## Abstract

The aim of this study was to examine the oxidation of selected plant oils in concentrated beverage emulsions colored with natural β-carotene. Carotenoid preparations obtained from carrots were dissolved in cold-pressed linseed oil, refined canola oil, and refined palm olein. Oxidative stability of the lipids was examined with and without addition of the pigment to the oil/water (O/W) emulsion. Carotenoid/lipid hydro peroxide (LOOH) concentration was evaluated using two different methods: LOOH + Fe^2+^ reaction connected with a colored complex of ammonium thiocyanate determined with the help of a spectrophotometer, and LOOH determined with the help of a chemiluminometer. It was shown that oxidation rate of lipids in the O/W emulsions strongly depended on chemical composition of the lipid fraction (type of oil used). Presence of the carotenoid pigment increased the rate. Therefore, if a carotenoid-containing emulsion is to be stable, it should be based on oils of a high oxidative stability.

## Introduction

Stability of concentrated beverage emulsions is related to changes in their physicochemical properties during storage. Physical changes related to mobility of an emulsion dispersed phase degrade it. Each system composed of many small droplets needs a higher total surface energy than a corresponding system composed of fewer larger droplets. Since every physical system tends to attain the lowest possible energy level, over a long period, dispersed phase droplets undergo the creaming process. The process of physical destabilization of concentrated beverage emulsions is extensively described in the literature [1–8].

Apart from physical changes, some chemical transformations of emulsion components also take place [9]. Beverage emulsions based on vegetable oils (a non-polar phase medium) are composed of oils and dyes soluble in both water and lipids. Chemical changes occurring in emulsions are mainly related to oxidation of the lipid phase and the lipid-soluble components. The emulsion structure impacts the rate of the degradation.

The interfacial layer around every single droplet of the dispersed phase is composed mainly of molecules of a compound showing some surface activity [10]. The layer may also contain molecules of water, lipids, and compounds absorbed from the emulsion system (e.g. metal ions). The interfacial layer plays a key role in controlling rate of oxidation running within the emulsion [9, 10]. Decker [11] and Bing et al. [10] have demonstrated considerable differences in oxidation processes occurring in systems composed mainly of pure lipids and in emulsions. The differences reflected such factors as distribution of lipid molecules inside the emulsion, physical separation of individual emulsion constituents between different emulsion regions, and possible interactions between the compounds. Interfacial layers may also affect the oxidation of lipid droplets. Such differences are, however, observed only in oil/water (O/W) emulsions. In W/O systems where the lipid phase is directly exposed to the atmosphere (oxygen), the oxidation process proceeds basically as in pure lipids.

The most important difference between oxidation processes running in bulk lipids and in O/W emulsions concerns the transport of oxygen into the lipid [10]. For bulk lipids the air-borne oxygen is in direct contact with the lipid surface and the transport is as simple as air $\to$ lipid. The lipid oxidation rate depends on the fatty acid unsaturation level, the coefficient of diffusion of oxygen into the lipid, temperature, pressure, and the presence of light [10]. Transport of oxygen in emulsified systems follows a much more complicated path: air $\to$ aqueous phase $\to$ interface $\to$ lipid droplet. Since the concentration of oxygen in aqueous phase is much lower (strongly dependent on temperature) than in air, the oxidation process proceeds differently [10]. The situation is additionally complicated if various emulsifiers are used to stabilize the emulsion: the lipid oxidation rate may depend significantly on the type of the emulsifier used [10, 12–16]. Emulsifiers may affect the transfer of lipids from the aqueous phase into droplets [10] and that way may reduce the lipid oxidation rate. Lipids dispersed in the aqueous phase have a considerably larger contact surface and one might suspect that their oxidation would proceed at an elevated rate [10, 17–20]. The interfacial layer provides a physical barrier for the emulsion constituent to diffuse from particular emulsion regions. It was observed that even a small quantity of emulsifier produced interfacial layers on lipid droplet molecules, while the mass formed micelles around them. Those affected the transfer of various substances into the emulsion [9]. Interfacial layers in O/W emulsions are a medium, in which hydroxides interact with peroxidants present in the aqueous phase. That is why these phenomena are crucial to understanding the mechanism of oxidation in emulsion systems.

Lipid phase oxidation depends also on the dynamics of the emulsion system since the rate at which individual emulsion components may be inter-changed depends on the droplet collision frequency [9].

Various substances (sugars, polysaccharides, amino acids, proteins or salts) dissolved in the aqueous phase of the majority of edible emulsions may also strongly affect the oxidation process (increasing or decreasing its rate) [11, 16, 19, 21–23]. The rate of degradation of lipids and carotenoids in concentrated emulsions depends on the lipid phase dispersion level (at smaller droplet diameters the oxidation process proceeds faster due to the developed lipid surface) [24, 25], lipid concentration in the emulsion [14], type of the lipid used as emulsifier [14, 15, 26], temperature [27, 28], presence of light [27, 29] and pH of the aqueous medium [30].

Vegetable oils dispersed in emulsions are much more susceptible to oxidation than non-emulsified oils. The lipid oxidation process proceeds faster if the concentration of oil within the emulsion is lower [14, 21]. Emulsion stability depends largely on dispersion of the lipid phase in the aqueous medium. The smaller the droplet size, the more stable is the emulsion [31]. Emulsions with droplet diameters below 1 μm are extremely stable [4]. Literature data indicate that beverage emulsions stabilized with Arabic gum exhibit increased thermodynamic stability even at temperatures up to 100 °C [32]. Buffo et al. [33, 34] reported droplet diameters 0.75 … 0.92 μm in highly stable beverage emulsions stabilized with Arabic gum used as an emulsifier. Mirhosseini et al. [35] reported high stability exceeding 180 days in emulsions stabilized with 20 % of Arabic gum.

Carotenoids are easily oxidized by atmospheric oxygen due to their polyene-molecular structure. As a result, the color of the emulsion may vanish relatively rapidly [28, 36–38]. Moreover, carotenoids may also act as a pro-oxidative compound that lowers the resistance of oils to free oxygen [28, 39–44]. Differences in degradation rate of α- and β-carotene in different oil emulsions might be explained by differences in the oxidation rates of the oils.

Since stability of O/W emulsions—especially emulsions used in the food industry—has a very tangible practical aspect, gaining a further insight into oxidation processes running in emulsions and factors affecting those processes (in particular: oxidation rate) is a justified, well-worth effort. The aim of this study was to examine oxidation of selected plant oils in concentrated beverage emulsions colored with natural β-carotene.

## Materials and Methods

### Chemicals

Reagents/preparations used in this study (vendors in parentheses): Valgum, a mixture of different varieties of Arabic gum; Velrosin D, esterefied colophony used to increase lipid phase density (Valmar); cold pressed linseed oil (Szarlat, Poland); refined rapeseed oil (ZPT, Poland); refined palm olein (AJV, Poland); sodium benzoate, citric acid, *n*-hexane, acetone, methanol, chloroform, hydrochloric acid, ammonium thiocyanate, iron chlorine, di-methyl formamide/DMF and potassium hydroxide (POCH Gliwice, Poland); β-carotene standard and *tert*-butyl peroxide (Sigma-Aldrich, Germany).

### Samples

Six different concentrated beverage emulsions were prepared in ten replicates. Composition of the emulsions is shown in Table 1. Emulsions were refrigerated at 2 °C (±2 °C) before use. Rapid growth of molds was observed in emulsions stored at room temperature (RT).

**Table 1:** Composition of the concentrated beverage emulsions (%)

Component	E1	E2	E3	E4	E5	E6
Arabic gum	12					
Density increasing additive	5					
α- and β-carotene preparation	0.1			–	–	–
Cold pressed linseed oil	5	–	–	5	–	–
Refined rapeseed oil	–	5	–	–	5	–
Refined palm olein	–	–	5	–	–	5
Sodium benzoate	0.1					
Citric acid 2 mol/dm^3^	1					

### Aqueous Phase

Arabic gum and sodium benzoate were mixed with an appropriate volume of distilled water by a laboratory magnetic stirrer operated at 800 rpm for 30 min. The solution was acidified with 2 M citric acid up to pH 4 and left intact for 24 h at RT to allow the Arabic gum to rehydrate.

### Lipid Phase

Valrosin was shredded in a laboratory mill before use. The oil was mixed with an additive used to increase mixture density and mixed by hand with a glass stirring rod for 15 min. The mixture was heated in a water bath operated at 40 °C to fully dissolve the additive.

The carotenoid preparation was added to the oil/ballast additive mixture according to the recipe. The solution was heated in a water bath and mixed by hand until the dye crystals had fully dissolved. Then the solution was cooled and dispersed at RT.

### Pre-Emulsion

Oily phase was dispersed in the aqueous phase by intensive stirring with a laboratory stirrer operated at 1,500 rpm for 15 min.

### Homogenization

The pre-emulsion was homogenized with an APV Systems model APV 100 homogenizer. The homogenization process was carried out at RT in two steps, at 55 MPa, and at 18 MPa.

### Dispersed Phase Particle Size Distribution

The size of the particles in the dispersed phase was analyzed with the laser diffraction method using a Zeta Sizer 4 (Malvern) apparatus.

### Isolation of α- and β-Carotene from Carrot

Carotenoid preparation was obtained according to the procedure described previously [45].

### Determination of Carotenoid Contents

First, 100 μl of concentrated emulsion was transferred into a 15-ml Teflon centrifuge tube and mixed with 4.9 ml of distilled water. Then 5 ml of hexane:acetone mixture (1:1 v/v) was added to the tube, the tube was closed with a stopper, and the solution was mixed for 1 min. The tube was then centrifuged for 10 min at 4,000 rpm. Subsequently 3 ml of the hexane layer was transferred into a 10 ml measuring flask and diluted with hexane up to a 10-ml final volume. The total carotenoid contents was determined with a spectrophotometer operated at 450 nm.

### Spectrophotometric Determination of Lipid Hydro Peroxides

For the purpose of this study the modified methods reported by Hornero-Mendez’a et al. [46] and Szterk and Lewicki [47] were used. First, 100 μl of the emulsion was transferred into a 15-ml PTFE centrifuge tube and mixed with 4.9 ml of distilled water. Subsequently 5 ml of the acetone:hexane (1:1) mixture was added. Tubes were sealed with a stopper and shaken for 1 min. Tubes were centrifuged for 10 min at 4,000 rpm. Subsequently, 1 ml of the hexane layer was mixed with 4 ml of methanol:chloroform:hydrochloric acid (1:1:0.012) solution, then with 100 μl of 30 % ammonium thiocyanate aqueous solution. Finally, 100 μl of 0.4 % ferric chloride was added. Absorbance at *λ* = 480 nm was measured 5 min after the ferric chloride addition. Measurements were made against a blank sample prepared without any emulsion. Hydro peroxide concentrations were calculated using a calibration curve plotted with *tert*-butyl peroxide.

### Chemiluminometric Determination of Peroxides

First, 100 μl of the emulsion was transferred into a 15-ml PTFE centrifuge tube and mixed with 4.9 ml of distilled water. Subsequently 5 ml of the acetone:hexane (1:1) mixture was added. Tubes were sealed with a stopcock and shaken for 1 min. Tubes were centrifuged for 10 min at 4,000 rpm. 200 μl of the hexane layer was transferred into a chemiluminometer vial [47]. The reaction was initiated with introduction of 2 ml of saturated potassium hydroxide in DMF (5 g KOH/150 ml DMF) through a instrument capillary. Changes in chemiluminescence were measured for 3 min after adding the reagent.

### Statistical Analysis

Six different concentrated beverage emulsions were prepared in ten replicates (*n* = 10). Results were processed using standard tools of the Microsoft Office 2010 suite. The data were analyzed using STATISTICA 9.0 computer software and the Duncan multiple range test was used within the ANOVA procedure to compare differences among means from replicated experiments. Significance was defined at *P* < 0.05 level. In the figures, we present the results of statistical analysis in the form of error bars.

## Results and Discussion

### Particle Size Distribution

**Fig. 1:**
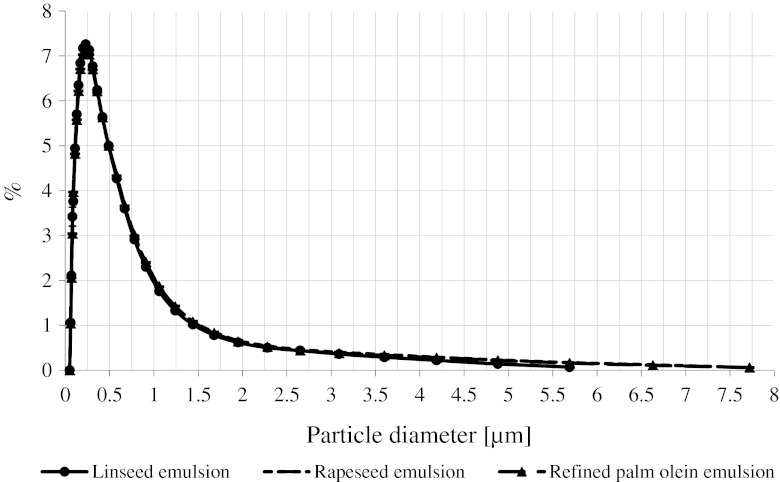
Distribution of the droplets size in the concentrated beverage emulsions prepared without carotenoid additive

Figure 1 shows the percentage distribution of the size of droplets dispersed in emulsions made of cold pressed linseed oil, refined rapeseed oil, and refined palm olein. Diameters related to volume *D*(4,3) were equal to 0.39/0.42/0.41 μm for the linseed oil/rapeseed oil/palm olein emulsion, respectively (no statistically significant differences at *P* > 0.05). Median diameter related to volume *D*(*v*, 0.5) was 0.24 μm in each case.

**Fig. 2:**
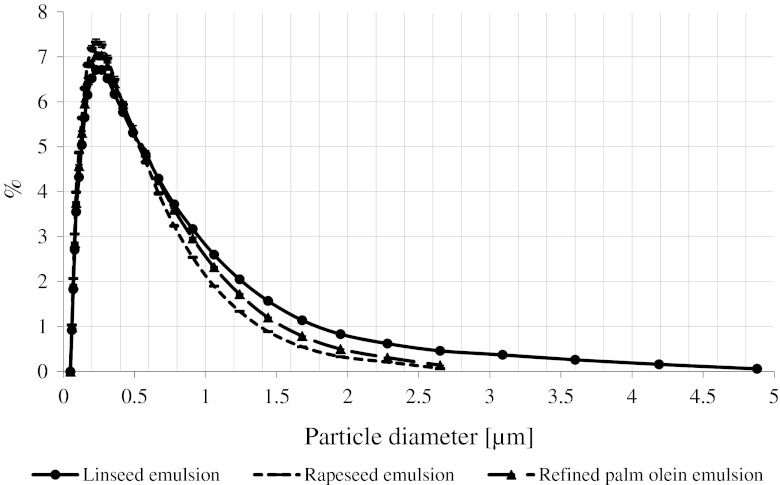
Distribution of the droplets size in the concentrated beverage emulsions prepared with α- and β-carotene additive

Figure 2 shows the percentage distribution of the size of droplets dispersed in emulsions prepared with the addition of fixed amounts of α- and β-carotene. Diameters related to volume *D*(4,3) were equal to 0.43/0.34/0.36 μm for the linseed oil/rapeseed oil/palm olein emulsion, respectively. Median diameters related to volume *D*(*v*, 0.5) were equal to 0.27/0.24/0.25 μm for the linseed oil/rapeseed oil/palm olein emulsion, respectively. Values for the rapeseed oil and palm olein emulsions did not differ statistically (*P* > 0.05). However, a statistically significant difference was observed in the case of the linseed oil emulsion. Generally, the low diameter of oil droplets *D*(*v*, 0.5) = 0.24 … 0.27 μm ensured high thermodynamic stability of the emulsions. No creaming was observed for 12 weeks of storage.

Most probably, the observed differences in droplet sizes in emulsions with and without some carotenoid added may be attributed to some changes in the lipid phase physical and chemical properties caused by the carotenoids. Carotenoids exhibit various polarities and affinity to oils. However, the differences in droplet sizes in emulsions with dye addition were not high enough to significantly decrease the stability of the studied emulsions.

### Carotenoid Concentration

**Fig. 3:**
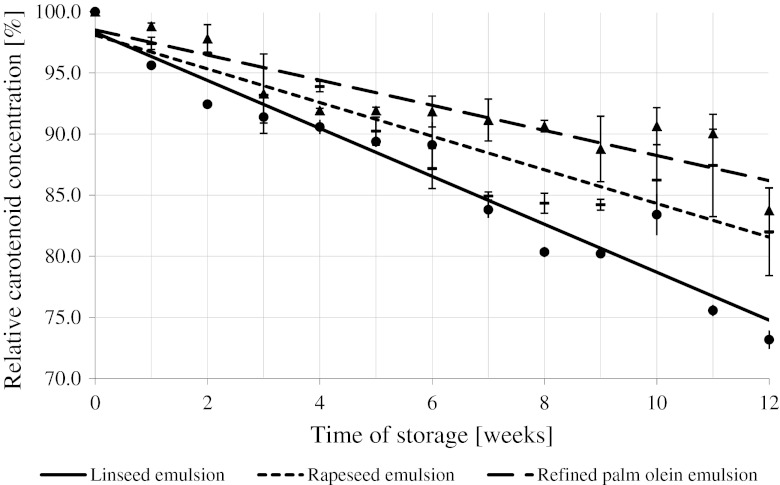
Changes of the total carotenoid contents in the concentrated emulsions stored at +2 °C

Figure 3 shows the total carotenoid contents in the concentrated emulsions during their 12-week long storage. The highest slope of the dye decay curve (tan *α* = −1.96) was observed for the cold-pressed linseed oil emulsion. During 12 weeks of storage, we observed a 26.8 % drop in the carotenoid contents in that emulsion. Rapeseed oil/palm olein-based beverage emulsions were significantly more stable: the slope representing the decay of the carotenoid during storage was equal to tan *α* = −1.31/−1.02, respectively. Nevertheless, the carotenoid contents dropped by 18 % after 12 weeks of storage. One can calculate the time necessary to decompose 50 % of the carotenoids as 24.7/32.7/47.5 weeks from the day the emulsion was prepared for linseed/rapeseed/palm emulsions, respectively. These times are statistically different (*P* < 0.05).

In most cases, the process of oxidative degradation of carotenoids dissolved in emulsified and non-emulsified lipids proceeds at a fixed rate [48, 49]; such a fixed rate was also observed in this study.

The observed drop in carotenoid concentration during emulsion storage at 2 °C may be attributed (according to References [28, 29, 48, 50, 51]) to oxidation of the whole lipid phase (both carotenoids and oils). The conducted study revealed that the rate of carotenoid oxidation is lower if oils used to prepare the emulsion are more resistant to oxidation.

### Peroxide Concentrations

**Fig. 4:**
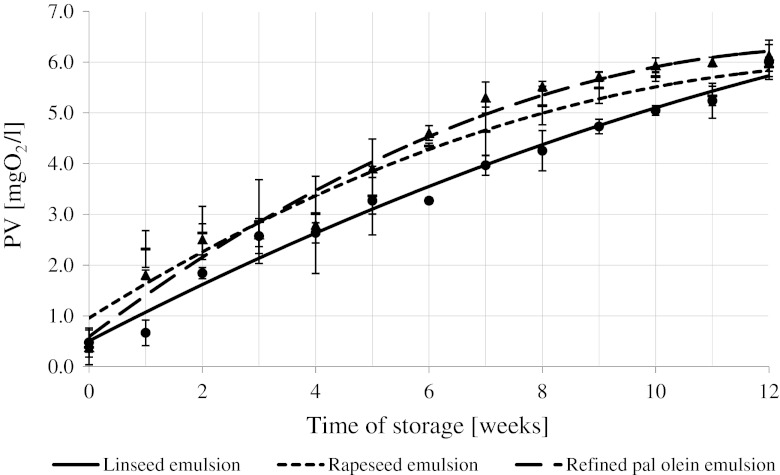
Changes in the peroxide value of the concentrated beverage emulsions within 12 weeks of storage at +2 °C

**Fig. 5:**
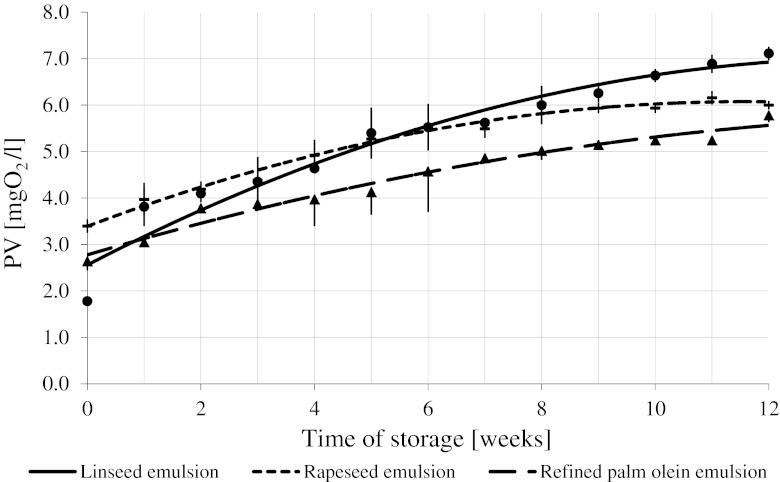
Change in the peroxide value of the concentrated beverage emulsions prepared with α- and β-carotene addition stored for 12 weeks at 2 °C

Figures 4 and 5 show time evolution of concentration of lipid hydro peroxides (LOOH) during storage of emulsions prepared with and without α-/β-carotene additives, respectively. A statistically significant increase in LOOH concentration was observed: the smallest one in linseed oil-based emulsions, the largest one in rapeseed oil- and palm olein-based emulsions. The concentration was increasing at some statistically slower rate in linseed emulsions, and at a higher rate in rapeseed/palm emulsions. No statistically significant difference (*P* > 0.05) between LOOH concentration increase rate in rapeseed and palm emulsions was observed. After 12 weeks of storage the LOOH concentration increased by approximately 6 mg O_2_/l for all studied emulsions. A statistically significant increase in LOOH value during 12 week-long refrigerated storage period was also observed in emulsions with a fixed amount of carotenoid preparation added. Similar pattern was observed by Szterk and Lewicki [47] in their study of pure oil oxidation: LOOH concentration increased more during palm olein and rapeseed oil oxidation that during linseed oil oxidation. Similar results were observed in concentrated beverages containing α- and β-carotene, stored at 2 °C. The somewhat slower increase in the LOOH concentration in linseed emulsions is most probably caused by the same phenomena as that observed during pure oil oxidation at 70 °C.

The rate at which lipid hydroperoxide is formed is slower than the rate at which molecules react between themselves and with other products of lipid auto-oxidation. Reactivity of LOOH formed in rapeseed oil and palm olein is most probably slower (and consequently their concentration is higher) than reactivity of LOOH formed in linseed oil due to the fact that composition of fatty acids in the former oils is more favorable [52]. On the other hand, rapeseed oil has the highest contents of tocopherols and should exhibit the highest oxidative stability [53]. The observed changes in LOOH concentration in emulsions prepared with the carotenoid preparation additive (Fig. 5) support that hypothesis.

The LOOH concentration increased at the highest rate in linseed/rapeseed oil and at the slowest rate in palm olein in agreement with carotenoid stability (Fig. 3). The type of oil used to prepare emulsions strongly affects carotenoid degradation and the LOOH formation process. Results obtained for carotenoid-spiked emulsions indicate that both emulsified and non-emulsified lipids are subject to the same oxidative processes.

The peroxide content is positively correlated with oxidative susceptibility of oils used to prepare the emulsions. α- and β-carotene added to an emulsion unify the process of oxidation running in the emulsion. Carotenoids are significantly more prone to oxidation than fatty acids bound in triacylglycerides [49]. Consequently, carotenoid hydro-peroxides (of similar reactivity) dominated the emulsion lipid phase. Therefore, it seems that the rate of oil oxidation determines the LOOH value in the lipid phase of the emulsion containing carotenoids. The presence of carotenoids decreased the rate at which LOOH was produced during storage (similar observations were reported by Kiokias and Gordon [49]). The most commonly encountered explanation involves the anti-oxidative potential of β-carotene. It should be, however, kept in mind that carotenoids are more prone to oxidation than fatty acids and might show pro-oxidative potency due to the generation of free radicals that subsequently catalyze the oxidation of the oils [28, 43, 50, 54, 55].

### Chemiluminescence

**Fig. 6:**
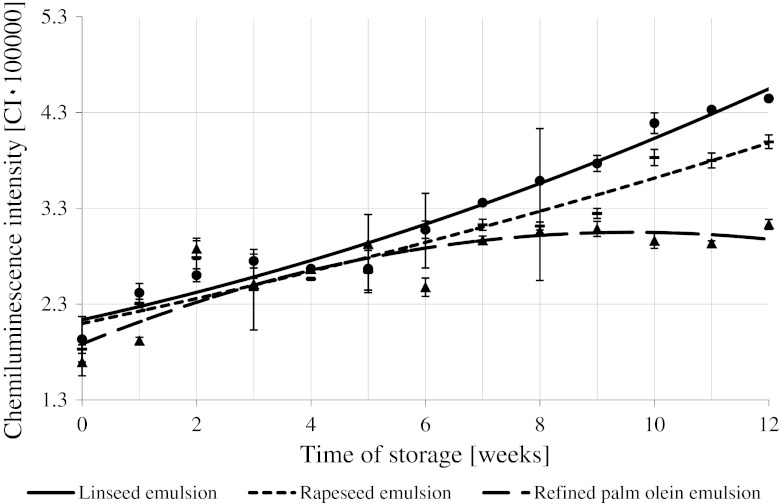
Changes in the intensity of the chemiluminescence (CI) of the concentrated beverage emulsions during storage at 2 °C

Figure 6 shows the time evolution of chemiluminescence intensity (CI) observed during 12 weeks of refrigerated storage of the studied emulsions. In each case, CI increased with storage time. However, CI dynamics depended on emulsion type: the CI changed more in linseed oil-based emulsions than in rapeseed oil-based ones, the changes were the smallest in emulsion containing palm olein. Differences between final CI intensities in various emulsions were statistically significant: CI ≈ 4.4/3.9/3.1 × 10^5^ for linseed oil/rapeseed oil/palm olein emulsions, respectively.

**Fig. 7:**
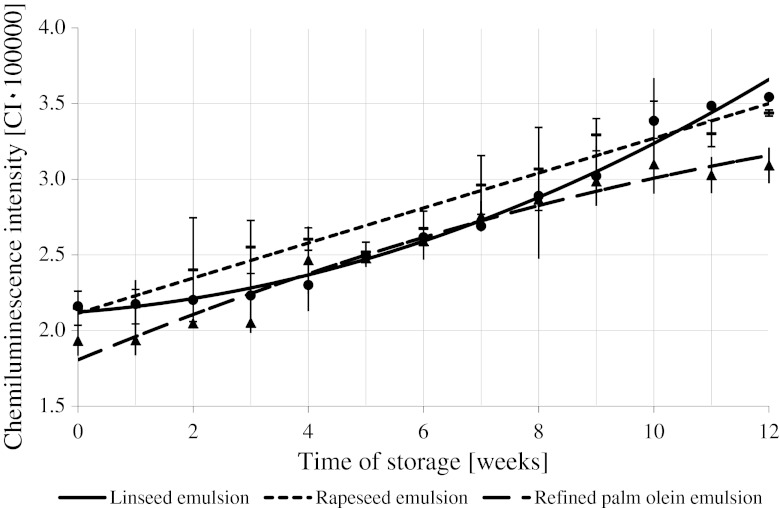
Changes in the chemiluminescence intensity of the concentrated beverage emulsions prepared with α- and β-carotene addition stored at 2 °C

Figure 7 shows the time evolution of CI observed during 12 week of refrigerated storage of the studied concentrated beverage emulsions containing a fixed amount of carotenoid preparation. Within the storage time, a statistically significant increase in CI was observed in all cases. CI increased at the highest rate in the rapeseed emulsion, at a somewhat slower rate in the linseed emulsion, and at the slowest rate in the palm olein emulsion. After 12 weeks of storage, the highest (statistically identical) CI values were determined for the linseed and rapeseed emulsions (≈3.4 and 3.5 × 10^5^, respectively). A statistically lower CI ≈ 3.1 × 10^5^ value was observed only for the palm olein-based emulsion.

The chemiluminescence reaction applied for the purpose of this study was characterized by a strong photon emission. In our previous papers, we showed that a strong linear correlation between the number of emitted light photons and the peroxide value of the vegetable oils oxidized at 70 °C existed [47, 52, 53].

The results of chemiluminescence determinations obtained for the concentrated beverage emulsions are similar to those obtained for the peroxide value evaluated with a standard method. Chemiluminescence increased during storage of emulsions prepared both with and without carotenoids. The CI value—just like the peroxide value—increased at a slower rate and reached a smaller final value in emulsions with dye than in emulsions without carotenoids.

## Summary

The oxidative stability of carotenoids dissolved in the emulsion lipid phase depends on the type of oil used to prepare the emulsion. Results of this study indicate that rapeseed oil and palm olein may be a better choice than linseed oil if stable concentrated beverage emulsions are to be prepared. The chemiluminescence technique/reaction proposed in this study seems to be a versatile tool to monitor the degradation of lipids in the emulsified phase. Results obtained by means of lipid hydro-peroxide determination agreed with results obtained with the faster and simpler proposed chemiluminescence-based method. The process of oxidation of lipid fractions in O/W emulsions seems to run linearly with time. The rate depends on the composition on the lipid phase; carotenoid additives increase the rate. If a carotenoid-containing emulsion is to be stable, it should be based on oils with a high oxidative stability.
